# Supplementary material for: Loss of glutamate transporter eaat2a leads to aberrant neuronal excitability, recurrent epileptic seizures, and basal hypoactivity
Source: Glia. 2021 Oct 30;70(1):196–214. doi: 10.1002/glia.24106 (PMC9297858; doi:10.1002/glia.24106)
Supplement: Supplementary file 6 — APPENDIX S1: Supporting Information [file GLIA-70-196-s003.pdf]

SUPPORTING INFORMATION

Loss of glutamate transporter *eaat2a* leads to aberrant neuronal excitability, recurrent epileptic seizures and basal hypoactivity

Adriana L. Hotz, Ahmed Jamali, Nicolas N. Rieser, Stephanie Niklaus, Ecem Aydin, Sverre Myren-Svelstad, Laetitia Lalla, Nathalie Jurisch-Yaksi[*], Emre Yaksi[*] & Stephan C.F. Neuhauss[*,§]

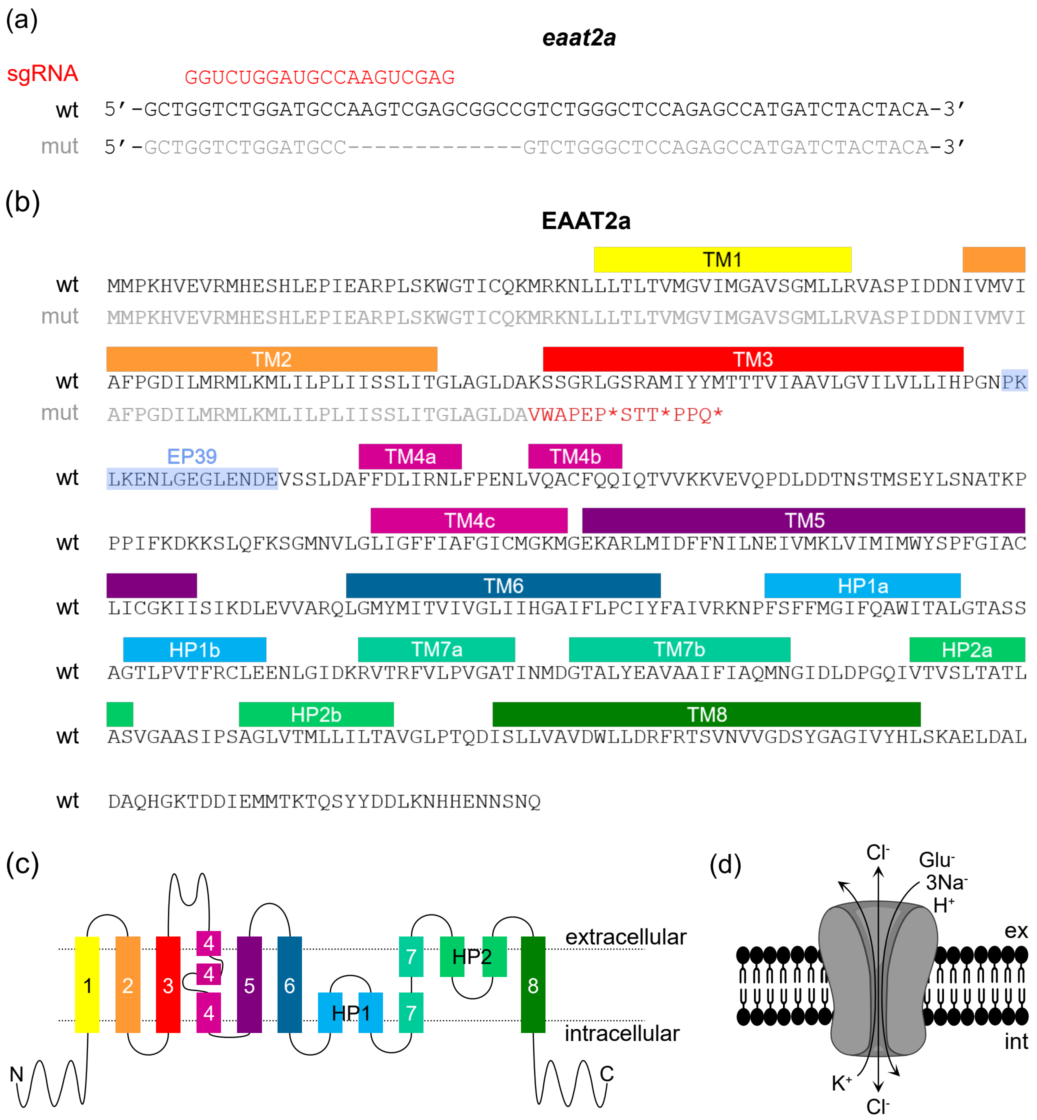

Supplementary Figure 1: Wild-type and mutant *eaat2a* sequence.

(a) Genomic DNA sequence of wild-type (black font) and mutant (gray font) *eaat2a* harboring a -13 base pair deletion. Target sequence of the single guide RNA used is represented in red font.

(b) EAAT2a wild-type (black font) and mutant (gray font) protein sequence with corresponding transmembrane domains (TMs) and hairpins (HPs) labelled. The -13 bp mutation leads to three premature stop codons (*) within TM3 at amino acids 109, 113 and 117. EP39 (epitope 39) highlights the anti-EAAT2a antibody recognition site.

(c) Two-dimensional schematic representation of EAAT2a protein structure. Color code for TM and HP labelling corresponds to color code in (b).

(d) Stoichiometry of EAATs.

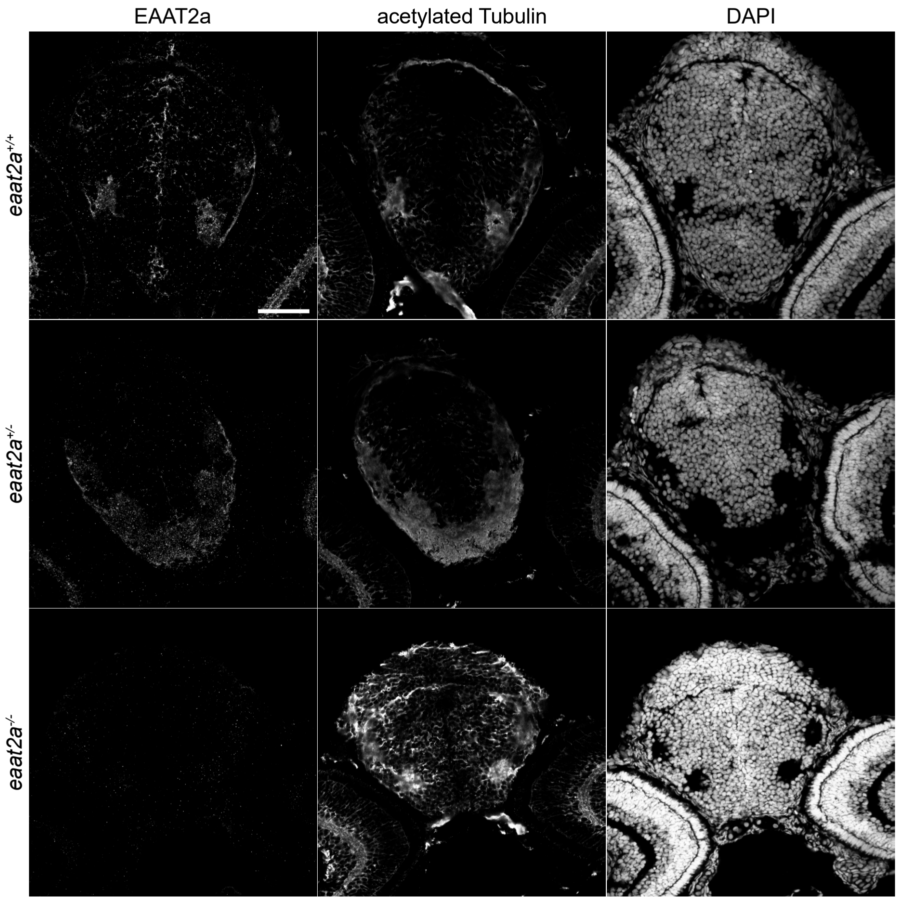

Supplementary Figure 2: Confirmation of EAAT2a protein knockout.

Immunostaining of EAAT2a and acetylated tubulin (acT) together with DAPI on cryosections of 3 dpf *eaat2a*$^{+/+}$ (top row), *eaat2a*$^{+/-}$ (middle row) and *eaat2a*$^{-/-}$ (bottom row) larvae. Scale bar is 50 µm.

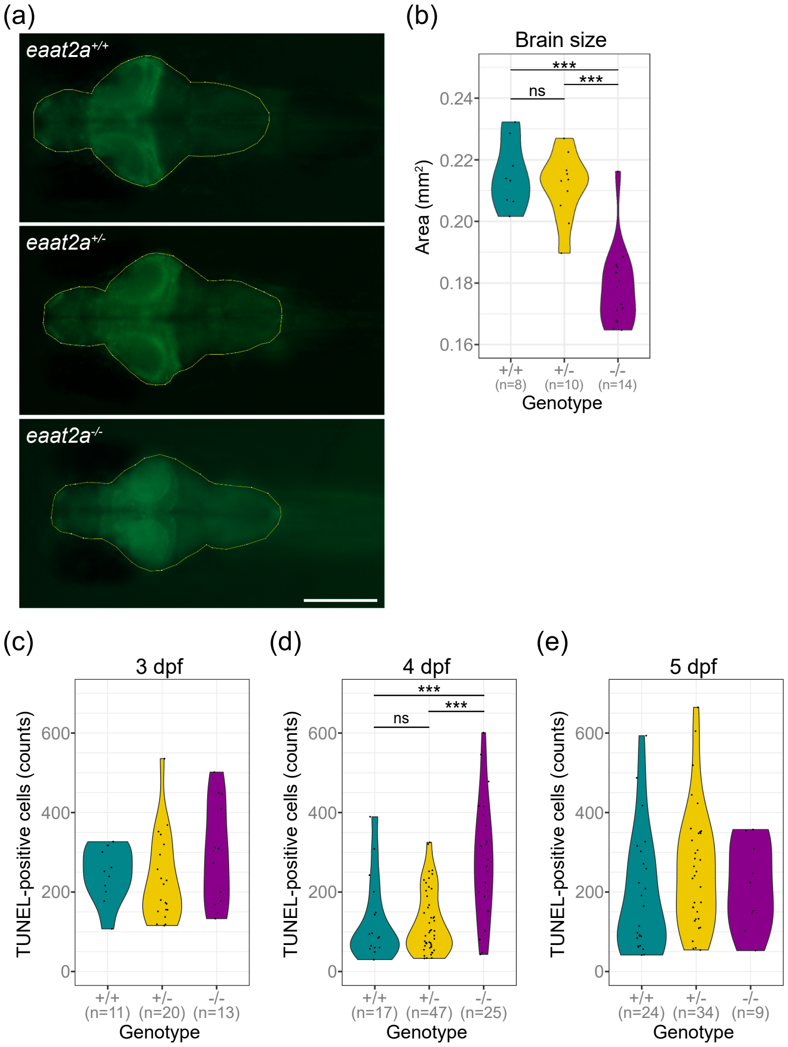

Supplementary Figure 3: In *eaat2a*$^{-/-}$ mutants, overall brain size was decreased at 5 dpf and apoptosis transiently increased at 4 dpf.

(a) Representative wide-field microscopy images of *eaat2a*$^{+/+}$ (top), *eaat2a*$^{+/-}$ (middle) and *eaat2a*$^{-/-}$ (bottom) larvae. Yellow selection shows region of interest used to measure total brain area by Fiji ImageJ (National Institutes of Health). Scale bar is 250 µm.

(b) Brain area was reduced at 5 dpf in *eaat2a*$^{-/-}$ mutants compared to their *eaat2a*$^{+/+}$ (p = 6.4e-4) and *eaat2a*$^{+/-}$ (p = 6.0e-4) siblings, while the area of *eaat2a*$^{+/-}$ was comparable to *eaat2a*$^{+/+}$ (p = 0.71)

(c-e) The number of TUNEL-positive cells in the brain of *eaat2a*$^{-/-}$ mutants was increased at 4 dpf compared to their *eaat2a*$^{+/+}$ (p = 3.9e-4) and *eaat2a*$^{+/-}$ (p = 5.0e-5) siblings (d), but similar at 3 dpf (c, $p_{+/+}$ = 0.56, $p_{+/-}$ = 0.44) and 5 dpf (e, $p_{+/+}$ = 0.62, $p_{+/-}$ = 0.61). The number in *eaat2a*$^{+/-}$ was comparable to *eaat2a*$^{+/+}$ in all stages (p_{3dpf} = 0.65, p_{4dpf} = 0.77, p_{5dpf} = 0.16).

Significance levels: ***$p \leq 0.001$, ns = not significant ($p > 0.05$), Dunn Kruskal-Wallis multiple comparison test (p-adjustment: Benjamini-Hochberg method)

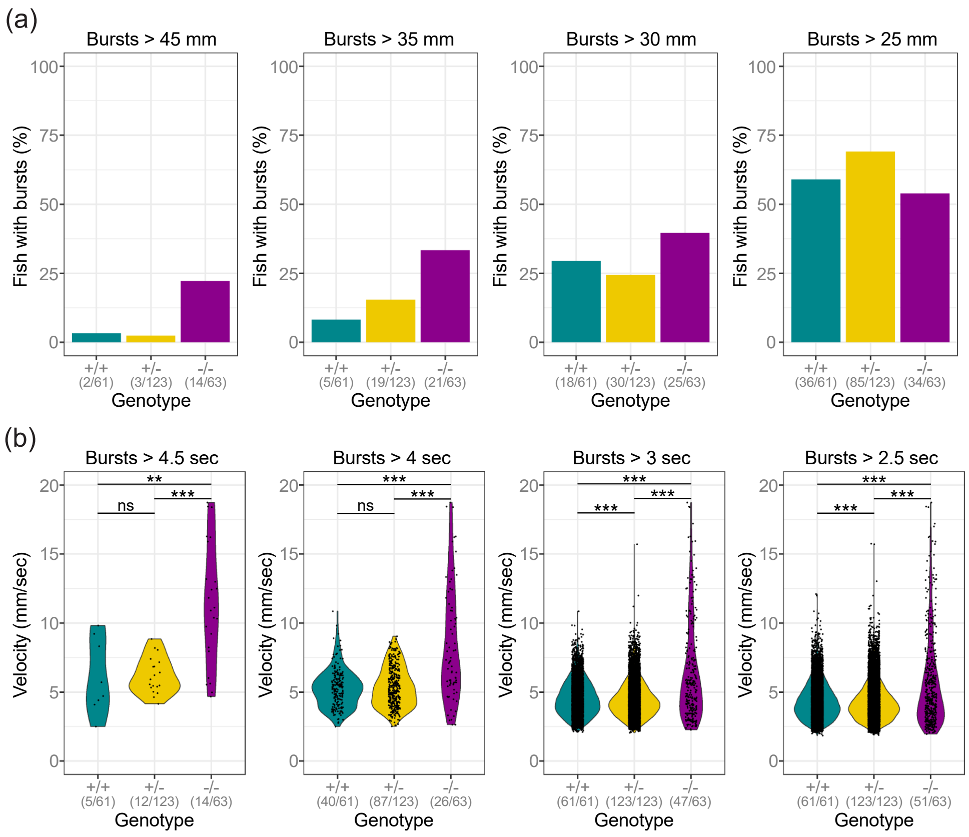

Supplementary Figure 4: Swim bursts per five-second integral in 5 dpf *eaat2a*$^{-/-}$ larvae were more frequent at higher thresholds.

(a) Proportion of fish showing one or more bursts bigger than threshold indicated in headings (mm).

(b) Velocity of all bursts lasting longer than threshold indicated in headings (sec). Significance levels: ***$p \leq 0.001$, **$p \leq 0.01$, ns = not significant ($p > 0.05$), two-sample Kolmogorov-Smirnov test.

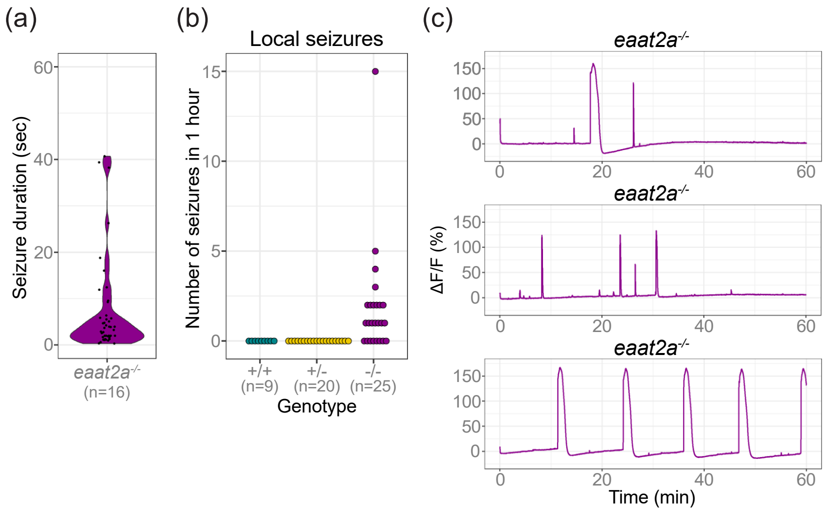

Supplementary Figure 5: 5 dpf *eaat2a*$^{-/-}$ mutants showed not only global but also local seizures not recruiting neurons of the anterior forebrain and lasting less than a minute.

(a) Duration of local seizures present in *eaat2a*$^{-/-}$ mutants (median 3.4 sec (std ± 10.3 sec)).

(b) Number of spontaneous local seizures per animal during 60 minutes.

(c) Change in GCaMP5G fluorescence (ΔF/F) over time recorded across the entire brain of three representative *eaat2a*$^{-/-}$ mutant larvae displaying local and global (top), exclusively local (middle) or exclusively global (bottom) spontaneous seizures.

Primer name	Primer sequence (5' – 3')	Amplicon (bp)
eaat2a sgRNA sg1	GAAATTAATACGACTCACTATA**GGTCTGGATGCCAAGTCGAG** GTTTTAGAGCTAGAAATAGC	20
eaat2a sgRNA sg2	AAAAGCACCGACTCGGTGCCACTTTTTCAAGTTGATAACGGACT AGCCTTATTTTAACTTGCTATTTCTAGCTCTAAAAC	
eaat2a sense fw genotyping	GATGCAGTCGTATGGGAA	204
eaat2a antisense rev genotyping	CCTTCTCCCAGATTCTCC	

Supplementary Table 1: List of primers used for CRISPR sgRNA synthesis and target region amplification.

Gene	www.ensembl.org/index.html	Fw primer (5' – 3')	Rev primer (5' – 3')	Amplicon (bp)
g6pd	ENSDARG00000071065	CTGGACCTGACCTACCATAGCAG	AGGCTTCCCTCAACTCATCACTG	127
b2m	ENSDARG00000053136	GACAAAGAAGTTTAGTTGGGAGCC	GAATCCATCGCTCCATCG	76
eaat1b	ENSDARG00000043148	CTGGTTCAAGCCTGCACTCAAC	CTCCTGCGTAGCGTTCGTC	111
eaat2b	ENSDARG00000052138	GGAATCGAACTTGATCCTGGTC	ATGTCTTGAGTCGGCAGTCC	143
mglur3	ENSDARG00000031712	GTCTTGATGGCAGGAAACTCTACC	CCACTCCATCTCCATACGCATC	117
mglur5b	ENSDARG00000102067	CCTGGTCGGAGAGTTTCTGCTG	GCAGACTGCAGCTTTATGGTGATC	114
gad1b	ENSDARG00000027419	ACAACAATGAGCGCTTCACG	TGAGGATCTCCACCACTTCGAG	126
gabra1	ENSDARG00000068989	GGTCACCAATGCTCTGGCTG	GAGACCAGGCCTAAGACGATTG	130

Supplementary Table 2: List of primers used for qRT-PCR experiments

Figure	Genotypes or timepoints compared	Age (dpf)	Analysis	n	Z, V, W, t values	p-value
2b	+/- to +/+	3	Dunn Kruskal Wallis multiple comparison test with Benjamini-Hochberg correction	n(+/+) = 17<br>n(+/-) = 26<br>n(-/-) = 23	Z = -0.27	0.79
2b	-/- to +/+	3			Z = -4.6	5.5e-6
2b	+/- to -/-	3			Z = 4.9	3.5e-6
2b	+/- to +/+	4			Z = -0.20	0.84
2b	-/- to +/+	4			Z = -5.1	3.9e-7
2b	+/- to -/-	4			Z = 5.5	1.0e-7
2b	+/- to +/+	5			Z = 0.18	0.85
2b	-/- to +/+	5			Z = -5.2	3.7e-7
2b	+/- to -/-	5			Z = 5.9	8.8e-9
2b	+/- to +/+	6			Z = 0.067	0.95
2b	-/- to +/+	6			Z = -5.2	2.6e-7
2b	+/- to -/-	6			Z = 5.9	1.2e-8
2b	+/- to +/+	7			Z = 0.084	0.93
2b	-/- to +/+	7			Z = -5.2	2.7e-7
2b	+/- to -/-	7			Z = 5.9	1.1e-8
2c	+/- to +/+	6	Dunn Kruskal Wallis multiple comparison test with Benjamini-Hochberg correction	n(+/+) = 37<br>n(+/-) = 56<br>n(-/-) = 42	Z = 0.14	0.88
2c	-/- to +/+	6			Z = -0.71	0.71
2c	+/- to -/-	6			Z = 0.86	1.0
2c	+/- to +/+	7			Z = 0.068	0.94
2c	-/- to +/+	7			Z = -1.5	0.19

2c	+/- to -/-	7	Dunn Kruskal Wallis multiple comparison test with Benjamini-Hochberg correction	n(+/+) = 37<br>n(+/-) = 56<br>n(-/-) = 42	Z = 1.6	0.35
2c	+/- to +/+	8			Z = 0.060	0.95
2c	-/- to +/+	8			Z = -3.2	3.5e-3
2c	+/- to -/-	8			Z = 3.2	2.2e-3
2c	+/- to +/+	9			Z = 0.060	0.95
2c	-/- to +/+	9			Z = -3.2	3.5e-3
2c	+/- to -/-	9			Z = 3.2	2.2e-3
2c	+/- to +/+	10			Z = 0.11	0.91
2c	-/- to +/+	10			Z = -2.9	5.1e-3
2c	+/- to -/-	10			Z = 3.0	7.1e-3
2c	+/- to +/+	11			Z = -0.16	0.87
2c	-/- to +/+	11			Z = -3.1	6.8e-3
2c	+/- to -/-	11			Z = 2.9	5.8e-3
2g	+/+ & +/- to -/- (after stimuli)	5	Two-sided Wilcoxon rank-sum test	n(+/+ & +/-) = 5<br>n(-/-) = 8	W = 858	7.7e-8
3c	+/- to +/+	5	Dunn Kruskal Wallis multiple comparison test with Benjamini-Hochberg correction	n(+/+) = 61<br>n(+/-) = 123<br>n(-/-) = 63	Z = -1.2	0.23
3c	-/- to +/+	5			Z = -10.2	2.9e-24
3c	+/- to -/-	5			Z = 10.6	7.6e-26
3f	+/+ to +/-	5	two-sample Kolmogorov-Smirnov test	n(+/+) = 60<br>n(+/-) = 121<br>n(-/-) = 42	D = 0.034	0.20
3f	+/+ to -/-	5			D = 0.36	< 2.2e-16
3f	+/- to -/-	5			D = 0.35	6.7e-16
3j	*eaat2a*$^{-/-}$ to PTZ	5	Two-sided Wilcoxon rank-sum test	n_{PTZ} = 6 fish, 10 seizures;<br>$n_{eaat2a-/-}$ = 8 fish, 40 seizures	z = 2.2	0.026
3k	*eaat2a*$^{-/-}$ to PTZ	5			z = 3.7	2.2e-4
4j	+/- to +/+	5	Dunn Kruskal Wallis multiple comparison test with Benjamini-Hochberg correction	n(+/+) = 9<br>n(+/-) = 20<br>n(-/-) = 23	Z = 0.18	0.86
4j	-/- to +/+	5			Z = -3.6	4.1e-4
4j	+/- to -/-	5			Z = 4.9	2.7e-6
5e	basal to preictal	5	Wilcoxon signed rank test	n(+/+ & +/-) = 6<br>n(-/-) = 8	V = 18	1
5e	basal to ictal	5			V = 0	0.0078
5e	preictal to ictal	5			V = 0	0.0078
5f	basal to preictal	5	Wilcoxon signed rank test	n(+/+ & +/-) = 6<br>n(-/-) = 8	V = 17	0.95
5f	basal to ictal	5			V = 0	0.0078
5f	preictal to ictal	5			V = 0	0.0078
5g	+/+ & +/- to -/- (after stimuli)	5	Two-sided Wilcoxon rank-sum test	n(+/+ & +/-) = 5<br>n(-/-) = 5	W = 94	8.7e-4
5h	+/+ & +/- to -/- (after stimuli)	5			W = 95	6.7e-4
5i	+/+ and +/- to -/-	5	Welch two sample unpaired t-test	n(+/+ & +/-) = 6<br>n(-/-) = 8	t(7.9) = 3.3	0.011
5j	+/+ and +/- to -/-	5	Two-sided Wilcoxon rank-sum test	n(+/+ & +/-) = 6<br>n(-/-) = 8	W = 15339	0.0011
5k	+/+ and +/- to -/-	5			W = 44420	0.052
5l	+/+ and +/- to -/-	5			W = 42282	0.37
5m	+/+ to -/-: *eaat1b*	5	Welch two sample unpaired t-test	4 biol. samples each, n(+/+) = 28-59 (x̄ 48) n(-/-) = 24-56 (x̄ 46)	t(5.7) = -6.5	7.5e-4
5m	+/+ to -/-: *eaat2b*	5			t(6.0) = -0.074	0.94
5m	+/+ to -/-: *gad1b*	5			t(6.0) = -3.0	0.024
5m	+/+ to -/-: *mglur3*	5		3 biol. samples each, n(+/+) = 50-59 (x̄ 55) n(-/-) = 47-56 (x̄ 53)	t(5.9) = 0.19	0.86
5m	+/+ to -/-: *gabra1*	5			t(3.9) = 0.95	0.40
5m	+/+ to -/-: *mglur5b*	5			t(3.9) = 0.94	0.40

Supplementary Table 3: Statistical analyses

SUPPLEMENTARY METHODS

TdT-mediated dUTP-biotin nick end labeling (TUNEL)

Offspring of *eaat2a*$^{+/-}$ fish was raised in 1x 1-phenyl-2-thiourea (PTU). At 3, 4 and 5 dpf, larvae were anesthetized on ice and fixed in 4% paraformaldehyde (PFA) at 4°C overnight. After washing in 1x PBT (0.1% Tween20 (Sigma-Aldrich) in 1x PBS), larvae were dehydrated in series of increasing methanol concentrations in 1x PBT (25%, 50%, 75%, 100% methanol), stored at -20°C for at least 2h, rehydrated in reverse methanol series, and quickly washed in 1x PBT. Larvae were then digested with Proteinase K (30 µg/mL) for 50 min (3 dpf), 60 min (4 dpf), or 75 min (5 dpf) respectively. After digestion and brief wash in PBT, larvae were fixed first in 4% PFA at room temperature (RT), then in pre-chilled (-20°C) ethanol:acetic acid 2:1 for 20 min each. Washing PBT was then replaced by Equilibrium buffer (ApopTag Peroxicase In Situ Apoptosis Detection Kit S7100, MILLIPORE) for 1-2 hours at RT. After removing Equilibrium buffer, larvae were incubated in Reaction buffer and TdT enzyme according to the kit at 37°C overnight. The next day, larvae were washed first 3h in working strength Stop/Wash buffer from the kit at 37°C, then three times in 1x PBT at RT. After blocking at least 2 hours in blocking buffer (0.1 mg/mL bovine serum albumine, 5% normal goat serum in 1x PBT), larve were incubated in anti-DIG AP antibody (Roche, 1:5’000 in blocking buffer) at 4°C overnight. The next day, larvae were washed first ten times in 1x PBT, then in 0.1M Tris-HCl (Sigma-Aldrich) pH 9.5 with 0.1% Tween20, and finally in NTMT (0.1M Tris-HCl pH 9.5, 0.1M NaCl, 0.05M MgCl2, 0.1% Tween20, 0.001M Levamisol in ddH2O). Staining was performed in fresh NTMT containing 3.5 µL/mL 5-bromo-4-chloro-3-indolyl phosphate (BCIP, Roche) and 2.25 µL/mL nitro blue tetrazolium (NBT, Roche) for 15-25 minutes, then stopped by washing the larvae five times in 1x PBT. After stepwise glycerol series (25% and 50% in 1x PBT), larvae were kept at least overnight at 4°C in 70% glycerol in 1x PBT. Before imaging, larvae were incubated in 100% glycerol for at least 1 hour at RT. Z-stack images were aquired on an Olympus BX61 microscope using DIC optics and analysed by a minimum intensity projection using Fiji (ImageJ). Apoptotic cells were counted manually in a blinded fashion using the cell counter plugin of Fiji (ImageJ). Statistical analysis was done by Dunn Kruskal-Wallis multiple comparison test (p-adjustment: Benjamini-Hochberg method) using FSA package in R software version 3.6.0 with the RStudio version 1.2.1335 interface (R Core Team, 2019; RStudio Team, 2018). Imaged larvae were subsequently washed several times in 1x PBT and genotyped for *eaat2a*.
